# A Novel Design Framework for Structures/Materials with Enhanced Mechanical Performance

**DOI:** 10.3390/ma11040576

**Published:** 2018-04-09

**Authors:** Jie Liu, Xiaonan Fan, Guilin Wen, Qixiang Qing, Hongxin Wang, Gang Zhao

**Affiliations:** 1 Center for Research on Leading Technology of Special Equipment, School of Mechanical and Electric Engineering, Guangzhou University, Guangzhou 510006, China; jliu@gzhu.edu.cn; 2 State Key Laboratory of Advanced Design and Manufacturing for Vehicle Body, Hunan University, Changsha 410082, China; xiaonanfan@hnu.edu.cn (X.F.); qixiangcn@163.com (Q.Q.); wanghx@hnu.edu.cn (H.W.); gangzhao@hnu.edu.cn (G.Z.)

**Keywords:** design and fabrication framework, origami, topological design

## Abstract

Structure/material requires simultaneous consideration of both its design and manufacturing processes to dramatically enhance its manufacturability, assembly and maintainability. In this work, a novel design framework for structural/material with a desired mechanical performance and compelling topological design properties achieved using origami techniques is presented. The framework comprises four procedures, including topological design, unfold, reduction manufacturing, and fold. The topological design method, i.e., the solid isotropic material penalization (SIMP) method, serves to optimize the structure in order to achieve the preferred mechanical characteristics, and the origami technique is exploited to allow the structure to be rapidly and easily fabricated. Topological design and unfold procedures can be conveniently completed in a computer; then, reduction manufacturing, i.e., cutting, is performed to remove materials from the unfolded flat plate; the final structure is obtained by folding out the plate from the previous procedure. A series of cantilevers, consisting of origami parallel creases and Miura-ori (usually regarded as a metamaterial) and made of paperboard, are designed with the least weight and the required stiffness by using the proposed framework. The findings here furnish an alternative design framework for engineering structures that could be better than the 3D-printing technique, especially for large structures made of thin metal materials.

## 1. Introduction

The product design process is normally divorced from the manufacturing process, leading to extremely poor manufacturability, assembly and maintainability of the product. Thus, simultaneously consideration of the design and manufacturing processes is desired in actual engineering.

The specific functionalities and mechanical performances of a product need to be taken into account during the design process according to its service environment. This can be rather a simple task for structural optimization techniques [1]. Structural optimization methods can be broadly divided into three categories namely, size optimization [2], shape optimization [3,4], and topology optimization [5,6,7,8,9,10,11,12] methods. The main differences among the three methods are the design variables and the design freedom. Compared with the former two methods, topology optimization is a challenging and active research field that can produce various innovative candidates with expected mechanical properties. Since the inception of the homogenization method [5], this field has received a growing level of attention, emerging as a series of methods that include density-based methods, hard-kill methods, boundary-variation methods, and so forth. Among them, the solid isotropic material penalization (SIMP) method [5], the evolutionary structural optimization (ESO) method [7] and its improved version the bi-directional evolutionary structural optimization (BESO) method [13,14], and the level-set method [8,9,10] are recognized as the most widely used, and have been applied in various fields, covering aerospace [15], multifunctional materials [16,17,18], biomedical design [19,20], and uncertain design [21,22,23], etc.

Traditionally, engineers have utilized the reduction manufacturing method to fabricate engineering structures, which will significantly waste materials in most cases. To this end, 3D printing [24], as a kind of rapid prototyping technology, based on a digital model file and the use of powdered metal or plastic bonding material, using a layer-by-layer printing method to construct objects, has gained engineers’ attention recently due to its myriad merits, such as savings in materials, producing structures with highly complex geometries, and so on. Although 3D printing has been successfully applied in automotive, aerospace, medical industries, civil engineering, and other fields [25,26,27], it has its limitations at the present, i.e., being relatively expensive and inefficient, limited available material and manufacturing size, and having accuracy and quality problems. Hence, 3D printing cannot replace traditional manufacturing industry and reduction manufacturing method is still the mainstream. 

Alternatively, one structural/material can be created by using the origami technique [28,29,30]. Origami is an ancient art such that it transforms a flat sheet of paper into a finished sculpture through folding along predefined creases. Inspired by its compelling and extraordinary features, the origami technique has been imitated and utilized to design metamaterials [31,32], self-folding structures [33], sandwich structures [34], mechanisms [35], and energy-absorbing structures [36,37], etc. Research into topological design for rigid foldable origami structures [38,39] mainly determines where to put the crease lines on the initial flat plane on the basis of the displacement response. Size optimization for the origami-based structures has also been observed. However, layout design for origami-based structures to look for the optimal material distribution has rarely been studied. 

In this study, the virtues of topological design and origami techniques are combined to propose a novel design framework for structures and materials. This framework can provide a fast design method for structures/materials with predominantly mechanical performances, especially for large structures made from thin metal plates. Here, the design of simple origami-based cantilevers is first investigated, and then the framework is utilized to design more complex origami-based cantilevers, i.e., Miura-ori-based cantilevers. 

## 2. Materials and Methods

### 2.1. Testing

**Figure 1:**
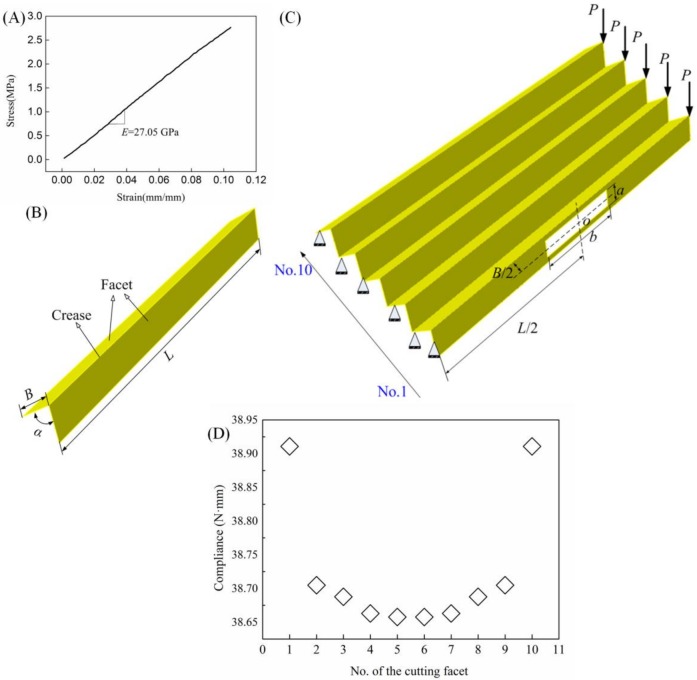
(**A**) The stress–strain curve for the paperboard; (**B**) the geometry for one unit cell of the origami with parallel crease lines; (**C**) the geometry, boundary, and loading conditions for the origami-based cantilever; and (**D**) the corresponding compliance of cutting holes in different facets.

Dynamic mechanical analysis is performed to characterize the paperboard, making up the origami-based cantilevers, by using a DMA Q800 machine (TA Instruments, New Castle, DE, USA). During the test, the temperature is about 24 °C and the humidity is close to 50% RH (Relative Humidity). The stress scan method is used, with force increasing from 0.05 to 10 N, with a logarithmic scale and 1 Hz force modulation. The stress–strain curve reported in Figure 1A indicates that the paperboard with thickness *t* = 0.32 mm is characterized by a Young’s modulus of *E*_p_ = 27.05 GPa (Figure 1A). It should be pointed out that for all the paperboard, a Poisson’s ratio of *υ*_p_ = 0.38 is assumed.

### 2.2. Geometrical Dimensions

Origami can be constructed by periodic arranged units. A unit consists of two identical rectangles (facet) with length, *L* = 250 mm, and width, *B* = 20 mm, and one crease (see Figure 1B). The dihedral angle is formed between two facets, *α* = 60°. For simplicity and without loss of generality, an origami with five units is considered and its facets are marked from No. 1 to No. 10 (see Figure 1C).

### 2.3. Finite Element (FE) Simulations

Finite element (FE) simulations are performed to investigate the deflection behaviors of the origami-based cantilevers by using the commercial package Abaqus/Standard 6.14. In all simulations, the models are divided with 3D shell elements (S4R). The size of the shell element is 2 × 2 mm. For the finite element model, its left boundary is fixed and five concentrated forces with equal magnitude, *P* = 5 N, are applied to its right side (see Figure 1C, in which the supporting marks shown symbolically indicate that the left end of the cantilever is fixed). The analysis method in Abaqus is static.

### 2.4. Topology Optimization

**Figure 2:**
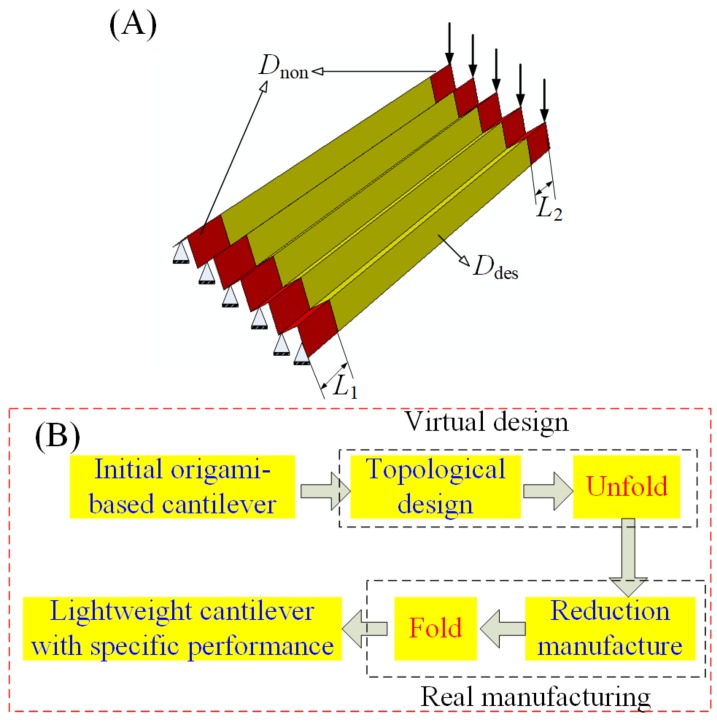
(**A**) The initial design and non-design domain for the origami-based cantilever; and (**B**) the proposed design and fabrication framework.

In actual engineering, a structure with a high stiffness–weight ratio is always sought by engineers, especially in the aviation field. Here, the computational approach, a continuum topology optimization method, is employed to design the layout of patterns to yield a structure with the aforementioned mechanical performance. In particular, the weight of the origami-based cantilever is minimized and its stiffness is restrained. For each design problem, two non-design domains, *D*_non_, highlighted in Figure 2A, are defined to maintain the integrity of the final optimal structure. The rest of the origami-based structure is defined as design domain, *D*_des_. This design domain can be occupied by solid elements or void elements.

Normally, a continuum topology optimization problem can be mathematically formulated as

(1) $$\begin{array}{ll} \mathrm{Minimize}: & f\left(\rho \right)\\ \mathrm{Subject}\;\mathrm{to}: & g_{i}\left(\rho \right)\leq 0,\;i=1,\ldots ,m\\ & h_{j}\left(\rho \right)=0,\;j=1,\ldots ,t\\ & \rho ={\left[\rho_{1},\ldots ,\rho_{e},\ldots ,\rho_{N}\right]}^{T}\\ & \;0<\rho_{\min}\leq \rho_{e}\leq 1 \end{array}$$

where f(⋅) is the objective function; g(⋅) and h(⋅) are the inequality and equality constraints, respectively; m and t represent the number of the inequality and equality constraints, respectively; $\rho_{e}$ stands for the design density which ranges from $\rho_{\min}$ (normally, $\rho_{\min}=0.001$) to 1; and N is the number of the elements occupying the design domain, *D*_des_. 

In this study, the minimum weight is desired and the maximal deflection is restrained. Since homogeneous material is used, minimizing structural weight is equivalent to minimizing the structural volume, and limiting the maximal deflection is equivalent to imposing a restriction on the structural compliance. The equality constraint is the structural static equilibrium equation. Thus, the objective function, f(⋅), the inequality constraint, g(⋅), and the equality constraint, h(⋅), can be respectively written as:

(2) f(ρ)=V(ρ)

(3) $$g\left(\rho \right)=C^{*}-C(\rho )\leq 0$$

(4) h(ρ)=P−K(ρ)U(ρ)=0

where V(⋅) is the volume; C(⋅) and $C^{*}$ are the structural compliance and a predefined limit for the structural compliance, respectively; and C(ρ)=PU(ρ). It is worth pointing out that the structural compliance presented here is equal to the strain energy of the structure. P indicates the vector of the applied load. K and U are the structural stiffness matrix and the vector of the displacement, respectively.

Using the penalty scheme [5], the Young’s modulus of the *e*th element can be expressed as:

(5) $$E_{e}=\rho_{e}^{p}E_{s}$$

where $E_{s}$ is the Young’s modulus of the solid element and p is the penalization factor, which usually has a value of 3.

The optimization model defined in Equation (1) is solved by a general optimization algorithm implemented in Abaqus. The filter technique [40] is employed to prevent the checkerboard problem. In order to ensure the optimal structure can be easily fabricated, the minimum and maximum thickness of the member size is controlled, which can be realized by using a geometric restriction in Abaqus. The optimization iteration procedure will be terminated when either the change of adjacent element densities or objective functions meet a prescribed convergence criterion.

### 2.5. Design and Fabrication Framework

The topological design method, in conjunction with origami techniques, forms a fast and efficient design and manufacturing strategy which can yield structures with the desired mechanical performances. The strategy mainly consists of four procedures, namely topological design, unfold, reduction manufacture, and fold, as shown in Figure 2B. Topological design and unfold procedures can be completed via computer, and are together named virtual design; while reduction manufacturing and fold are the real manufacturing procedures.

## 3. Results

### 3.1. Deflection Behavior of the Origami-Based Cantilever

To study the influence of removing materials on the deflection behavior of the origami-based cantilever, small holes are cut (equivalent to removing materials) in each facet. The holes may have different shapes and numbers. For the sake of simplification, the small rectangular hole at the middle of the each facet is considered. The rectangular hole has a length *b* = 60 mm and a width *a* = 12 mm (see Figure 1C).

The deflection behavior may be the primary consideration for the engineering structures. Generally, the deflection is determined by the structural stiffness in the elastic stage and the structural stiffness is the reciprocal of the structural compliance. Thus, structural compliance, *C*, is employed to characterize the deflection behavior of the origami-based cantilever.

Figure 1D shows the structural compliance of origami-based cantilevers with rectangular hole cutting in various facets, highlighted from No. 1 to No. 10, and shows that cutting materials from the structure may have great influence on structural stiffness. Hence, methods to restrict the deflection of the structure by removing materials quantificationally and directionally are needed to proceed. Topology optimization can control the structural maximal deflection at the same time as yielding a structure with the desired performance, i.e., the least weigh.

### 3.2. Design and Fabrication of the Origami-Based Cantilevers

**Figure 3:**
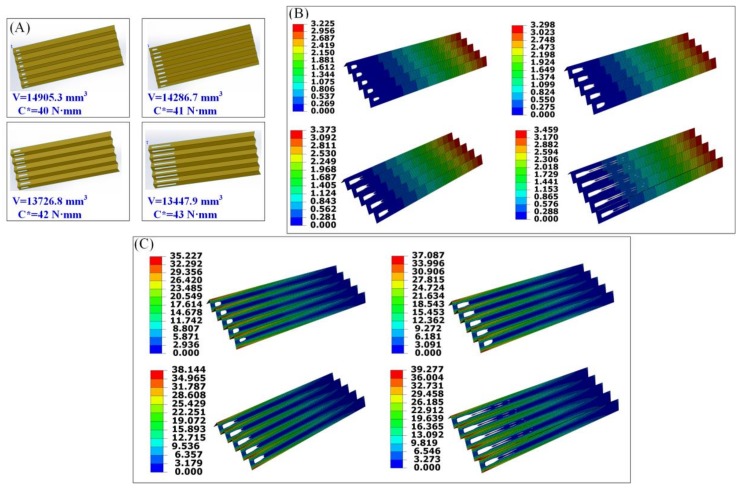

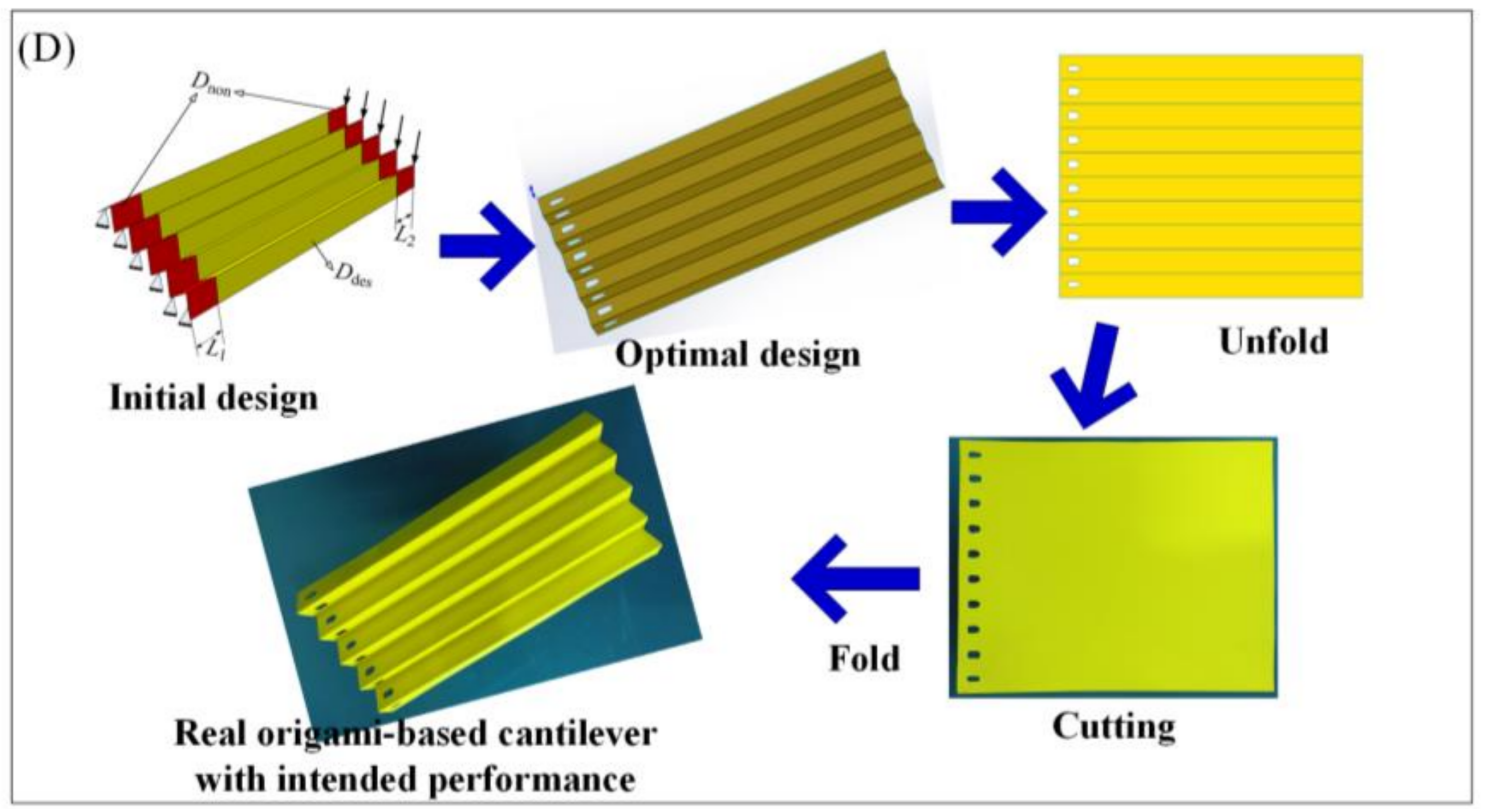
(**A**) Optimal designs for the origami-based cantilever under various constraints; (**B**) the corresponding displacements of the optimal solutions (unit: mm); (**C**) the corresponding stress distributions of the optimal solutions (unit: MPa); and (**D**) applying the proposed framework to the origami-based cantilever.

Figure 3A shows a series of optimal origami-based cantilevers obtained from the topology optimization with various limits for the structural compliance. The minimum and maximum thickness of the member size are controlled as 6 mm and 7 mm, respectively. The convergence criteria for the adjacent element densities and objective functions are 0.005 and 0.001, respectively. For the non-design domain, *L*_1_ = *L*_2_ = 6 mm. It can be clearly seen that the final layout and the final volume of the origami-based cantilever significantly differ from each other with different constraints. To be specific, the final volume is 14,905.3 mm^3^, 14,286.7 mm^3^, 13,726.8 mm^3^, and 13,447.9 mm^3^ when the predefined limit of the structural compliance is 40 N·mm, 41 N·mm, 42 N·mm, and 43 N·mm, respectively. It should be noted that the final volume of the origami-based cantilever is calculated based on the following equation:

(6) $$V=V_{total}-V_{removed}$$

where *V_total_* and *V_removed_* represent the total material volume of the paperboard making up the origami-based cantilever and the volume of the material removed (equals to the volume of the holes) from the initial design domain after performing the topology optimization algorithm, respectively.

It is easy to understand that more material will be removed from the initial design domain when the restraint for the structural compliance becomes larger, leading to a lighter weight of the origami-based cantilever. 

The corresponding displacements and stress distributions of the optimal solutions are depicted in Figure 3B,C, respectively. It is evident that the maximum displacement and stress become larger with the increase of the predefined limit for structural compliance. Specifically, the maximum displacement is 3.225 mm, 3.298 mm, 3.373 mm, and 3.459 mm, respectively; the maximum stress is respectively 35.23 MPa, 37.09 MPa, 38.14 MPa, and 39.28 MPa. It is easy to understand that the stiffness of the optimal origami-based cantilever gets smaller when increasing the predefined limit for the structural compliance. It can be also seen that stress concentrations occur at the areas of the creases, since the geometric shapes of these areas seem much sharper than those of the corners of the rectangular cut-outs, demonstrating that special attention should be paid to the creases when using the origami technique to fabricate the real origami-based structure. Nevertheless, the stress at the corners of the rectangular cut-outs is relatively larger than other areas except for the crease areas.

The first design is chosen to demonstrate our proposed design framework. Figure 3D presents the novel design and fabrication framework for the chosen origami-based cantilever. Apparently, the framework process includes the following steps: (1) establish the finite element model of the origami-based cantilever in Abaqus; (2) perform the continuum topology method to optimize the cantilever, and obtain the optimal design; (3) unfold the optimal design with specific patterns; (4) cut holes in the paperboard to obtain the patterns; (5) use the origami technique to fold the paperboard, achieving the real origami-based cantilever with its intended mechanical performance. It should be noted that the first two procedures are completed in Abaqus, the third procedure is finished in Solidworks, and the last two procedures are accomplished manually.

### 3.3. Design of Miura-Ori-Based Structures

**Figure 4:**
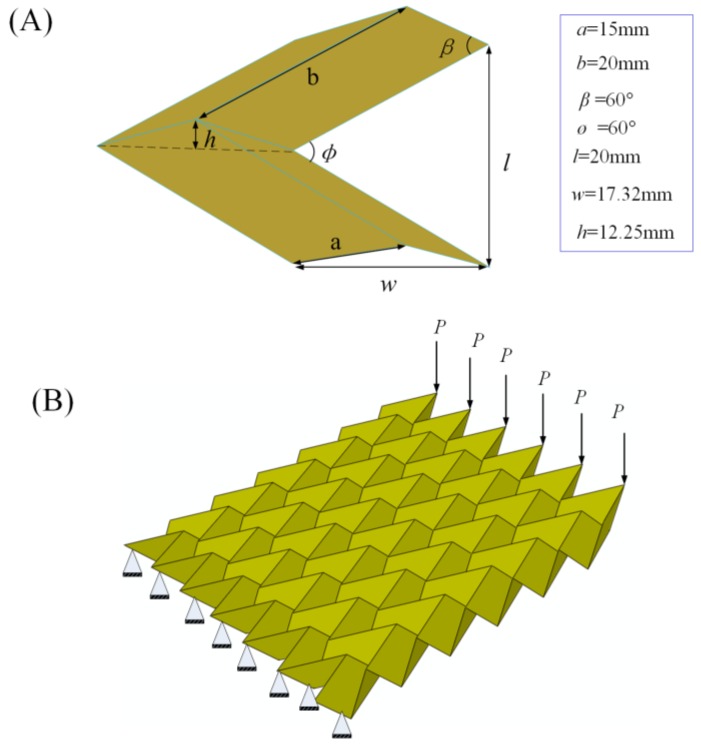
(**A**) The geometry for the unit cell of the Miura-ori; and (**B**) the geometry, boundaries, and loading conditions for the Miura-ori-based cantilever.

Using the proposed framework, we attempted to design more complex origami-based cantilevers. Here, the famous Miura-ori is selected. Figure 4 shows the geometry, boundaries and loading conditions for the Miura-ori-based cantilever. For the geometry, the Miura-ori consists of 36 unit cells. One unit cell (see Figure 4A) is formed by four parallelograms, and each parallelogram has sides *a* and *b* and acute angle *β*. The dihedral angle between two parallelograms is *ø*. The outer dimensions, like *l*, *w*, and *h*, can be determined by the geometric relation equations in reference [41]. The models are divided with 3D shell elements (S4R). Each element has a size of 2 × 2 mm. Its left boundary is fixed and six concentrated forces with equal magnitude, *P* = 5 N, are applied to its right side (see Figure 4B). It should be noted that a Miuta-ori-based structure is always considered to be rigid-foldable. Here, a facet as a deformable plate is considered, since the applied loads are assumed to be relatively small.

**Figure 5:**
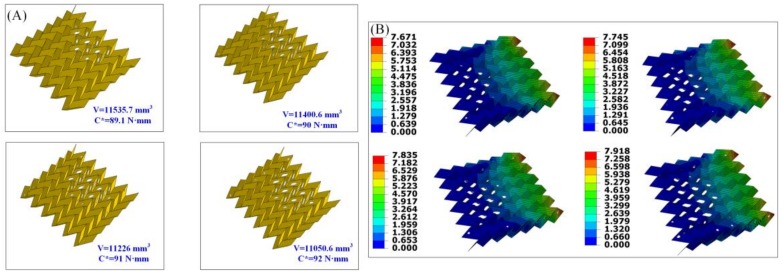

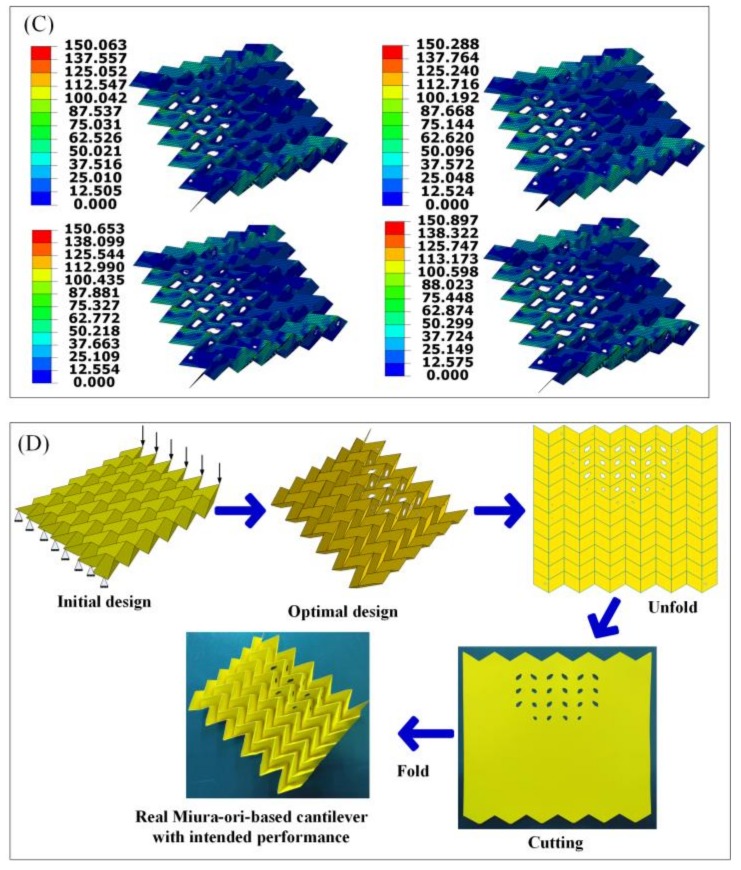
(**A**) Optimal designs for the Miura-ori-based cantilever under various constraints; (**B**) the corresponding displacements of the optimal solutions (unit: mm); (**C**) the corresponding stress distributions of the optimal solutions (unit: MPa); and (**D**) applying the proposed framework to the Miura-ori-based cantilever.

In the topological design, the minimum and maximum thickness of the member size is selected as 4.5 mm and 6 mm, respectively. The convergence criteria for the adjacent element densities and objective functions are set as 0.005 and 0.001, respectively. Figure 5A shows the optimal designs of the Miura-ori-based cantilevers. Similarly, more materials have been omitted from the initial design domain when the restraint for the structural compliance becomes larger, resulting in a lighter weight Miura-ori-based cantilever. Specifically, the final volume is 11,535.7 mm^3^, 11,400.6 mm^3^, 11,226 mm^3^, and 11,050.6 mm^3^ when the predefined limit of the structural compliance is 89.1 N·mm, 90 N·mm, 91 N·mm, and 92 N·mm, respectively.

Figure 5B,C separately present the corresponding displacement and stress distribution of the optimal outcomes in Figure 5A. As with the former design problem, the increasing values of the maximum displacement and stress can be clearly seen as the predefined limit for the structural compliance gets larger. Maximum stresses (stress concentration) occur at the areas of the vertices of the Miura-ori-based cantilever. Thus, these vertices should be carefully dealt with when creating the real structures.

The first design of the Miura-ori-based cantilever is selected to provide evidence of our proposed design framework. Figure 5D depicts the whole procedure of our framework. After establishing the finite element modeling of the Miura-ori-based cantilever, the design goal and constraints are given and the topological design is undertaken. The optimal design is obtained, and it is later unfolded in 3D modeling software, i.e., Solidworks. Then, holes (materials are removed by performing the topology optimization algorithm) are cut in the paperboard to get the patterns the same as that obtained from the 3D modeling software. Finally, the origami technique is exploited to fold the paperboard at the predefined crease lines, and the real Miura-ori-based cantilever with expected mechanical performances is achieved.

## 4. Discussion and Future Work

The design and fabrication of the origami-based cantilevers successfully demonstrate the feasibility and effectiveness of our proposed framework. The cantilevers are inspired by a simple origami with parallel creases and a complex Miura-ori. The framework is preferred for structures made of thin material. The desired mechanical performance is not restricted to the minimum weight and the limit of structural stiffness. Mechanical characteristics such maximum natural frequency, greatest stiffness, etc. can be also sought.

It should be noted that the optimal solution (Figure 3A and Figure 5A) cannot be ensured globally when using numerical topology optimization algorithms such as SIMP. This is the intrinsic characteristic of the algorithm. Optimal solutions are searched for by setting the structural stiffness requirements in advance. The designer can choose the favorite solution according to their demands. In the proposed design framework, the manufacturing cost may be mainly incurred through the third procedure, namely, cutting. Indeed, there is a possibility that the decrease of the volume is not acceptable in some cases in view of the increase of the manufacturing costs. Unfortunately, this work does not provide a quantitative index. Hopefully, a specific assessment will be proposed in our future work.

The maximum displacements of the optimal origami-based cantilevers (Figure 3B and Figure 5B) are larger with the increase of the predefined limit for structural compliance. Stress concentration normally arises at the areas of the creases or the vertices (Figure 3C and Figure 5C), revealing that these areas should be carefully dealt with when making real origami-based structures. The stresses at the corners of cut-outs are relatively smaller than the crease areas or the vertices and this may be because boundary-smoothing processing is performed to the cut-outs in Abaqus.

Here, materials are manually cut from paperboard. An alternative way to increase precision would be to use laser cutting that would produce accurate holes. To promote our framework for practical engineering applications, thin metal materials, e.g., aluminum, should be used to make the real origami-based cantilevers. Unfortunately, since the thickness of the metal affects the folding process, the real metal origami-based cantilevers can be difficult to make. To facilitate folding, at the crease lines the material may be locally thinned by means of chemical etching. It should be noted that the chemical-etching method will reduce the stiffness of the origami structure, which should be carefully considered. Real metal origami-based cantilevers could be manufactured by using a cold gas-pressure folding technique [39]. However, this method may only be suitable for small-scale structures as it is not an easy task to produce uniform pressure in order to fold large metal structures. Future work will attempt to use the framework to design and fabricate large engineering structures made of thin metal materials such as aluminium and steel.

## 5. Conclusions

In this paper, a novel framework for designing engineering structures/materials with intended mechanical performances by combining the compelling merits of topological design and origami techniques is proposed. The framework comprises four procedures, including topological design, unfold, reduction manufacturing, and fold. Specifically, the topological design method serves to optimize the structure to achieve the preferred mechanical characteristics, and the origami technique is exploited to make the structure in a way that can be rapidly and easily fabricated. One simple origami with parallel creases and one complex Miura-ori based cantilever are presented to validate the effectiveness of the proposed framework. The minimum weight of the structure with restrained stiffness is achieved via topological design, and the real structure is easily made by using the folding technique. This framework can be applied to design and fabricate large engineering structures made of thin metal materials, and is inexpensive and rapid compared with 3D-printing techniques. It should be noted that the proposed framework is not restricted to the design and fabrication of cantilevers; it can be applied to other kinds of structures as well.
